# miR-126 in Extracellular Vesicles Derived from Hepatoblastoma Cells Promotes the Tumorigenesis of Hepatoblastoma through Inducing the Differentiation of BMSCs into Cancer Stem Cells

**DOI:** 10.1155/2021/6744715

**Published:** 2021-10-29

**Authors:** Yu Hu, Hongyan Zai, Wei Jiang, Yuanbing Yao, Zhenglin Ou, Qin Zhu

**Affiliations:** Department of General Surgery, Xiangya Hospital, Central South University, 410008 Changsha, Hunan Province, China

## Abstract

**Background:**

Extracellular vesicles (EVs) can deliver miRNAs between cells and play a crucial role in hepatoblastoma progression. In this study, we explored the differentially expressed miRNAs related to tumor cell-derived EVs and the mechanism by which EVs regulate hepatoblastoma progression.

**Methods:**

Bioinformatics analysis was performed to explore the differentially expressed miRNAs between the hepatoblastoma and adjacent normal tissues. TEM, NTA, and western blotting were conducted to identify EVs. The expression of miR-126-3p, miR-126-5p, miR-30b-3p, miR-30b-3p, SRY, IL-1*α*, IL-6, and TGF-*β* was detected by RT-qPCR. Immunofluorescence (IF) was used to analyze the expression of PKH67, and flow cytometry was applied to assess the ratio of CD44+ CD90+ CD133+ cells. ELISA was used to evaluate the levels of IL-6 and TGF-*β*. A xenograft mouse model was constructed to detect the function of EVs with downregulated miR-126. IHC was performed to calculate *β*-catenin levels in tumor tissues.

**Results:**

miR-126 was upregulated in hepatoblastoma. EVs derived from hepatoblastoma cells significantly increased the ratio of CD44+ CD90+ CD133+ cells and increased the expression of IL-6, Oct4, SRY, and TGF-*β* in bone marrow mesenchymal stem cells (BMSCs), while EVs with downregulated miR-126 reversed these phenomena. miR-126 downregulation notably attenuated hepatoblastoma tumor growth and decreased the ratio of CD44+ CD90+ CD133+ cells and increased the expression of IL-6, Oct4, SRY, TGF-*β*, and *β*-catenin in tumor tissues of mice. Furthermore, EVs with downregulated miR-126 inhibited the differentiation of BMSCs into cancer stem cells.

**Conclusions:**

Exosomal miR-126 derived from hepatoblastoma cells promoted the tumorigenesis of liver cancer through inducing the differentiation of BMSCs into cancer stem cells.

## 1. Introduction

Hepatoblastoma is considered to be originated from undifferentiated liver cells in embryonic development [1, 2]. The incidence of hepatoblastoma is not very high, while over 80% of hepatoblastoma occurs under the age of 5 years [3]. In addition, hepatoblastoma brings about 80% of primary liver malignancies in children [4]. The first-line treatment for early-stage hepatoblastoma is complete resection of the liver [5, 6]. Nevertheless, preoperative chemotherapy is the major requirement for many patients with unresectable advanced-stage hepatoblastoma [7]. The developments of therapy intensification and surgical methods have led to the improvement of the survival rate among pediatric patients with hepatoblastoma over recent decades [7, 8]. However, the risk of toxicities from cumulative chemotherapy has been increasing [9]. Thereby, the patients with hepatoblastoma who are at high risk of a poor outcome should be identified during the therapy. At present, surgery is the major treatment for hepatoblastoma. However, the effect remains not ideal [10, 11]. Therefore, timely treatment of hepatoblastoma is of great significance for controlling this malignancy.

MicroRNAs (miRNAs) are noncoding small RNAs (21-24 nucleotides) which can modulate the levels of genes through mRNA translation inhibition or mRNA degradation [12, 13]. Thus, it can be considered that miRNAs are crucial mediators for many diseases. Previous findings revealed that miRNAs are involved in the progression of hepatoblastoma. For example, miR-29a could reverse circSTAT3-induced hepatoblastoma cell growth through targeting STAT3 [14]; Weiss et al. found downregulation of miR-483 could inhibit the proliferation of hepatoblastoma cells by suppressing the cell apoptosis [15]; downregulation of miR-203 could promote the angiogenesis of hepatoblastoma cells via activation of VEGFA [16]. Nevertheless, the functions of miRNAs in hepatoblastoma need to be further explored.

Previous reports have revealed that the association between tumor cells and stem cells in the tumor microenvironment can play a key role in modulating the development of cancer [17, 18]. In the tumor environment, BMSCs are often associated with tumor cells to regulate tumorigenesis and metastasis of cancers [19, 20]. In addition, it has been reported that BMSCs often differentiate into cancer stem cells during tumor metastasis and growth [21]. The correlation between tumor cells and BMSCs has been revealed in some cancers (gastric cancer, etc.) [22, 23]. Moreover, the previous study found that cancer stem cells- (CSCs-) and BMSCs-EVs reduce the expression of HCC miRNAs (miR148a, miR16, and miR125b) and induce epithelial-mesenchymal transformation [24]. However, the effect of miRNAs in the EVs of hepatoblastoma on the tumor microenvironment and its own progression is still unknown.

It is known that Wnt/*β*-catenin signaling plays a vital role in multiple cancers, including hepatoblastoma [25, 26]. For example, Sha et al. confirmed that the Wnt/*β*-catenin pathway could act as an effective therapeutic target in hepatoblastoma [27]; Regel et al. revealed that knockdown of SFRP1 promoted the tumorigenesis of hepatoblastoma through activation of Wnt/*β*-catenin signaling [28]. Thereby, we aimed to further explore the role of Wnt/*β*-catenin in hepatoblastoma.

In this study, we decided to identify and detect the differentially expressed miRNAs related to the EVs, which are associated with the development of hepatoblastoma. We hope this research would supply a novel method for hepatoblastoma treatment.

## 2. Materials and Methods

### 2.1. Cell Culture

Hepatoblastoma cell lines (huH6 and HepG2), normal liver cells (WRL68), and human bone marrow mesenchymal stem cells (BMSCs) were bought from ATCC (Manassas, VA, USA). Hepatoblastoma cells and WRL68 cells were maintained in DMEM (Thermo Fisher Scientific, Waltham, MA, USA) containing 10% FBS and 1% streptomycin and penicillin (Thermo Fisher Scientific) in an incubator (37°C, 5% CO_2_). BMSCs were maintained in *α*-MEM (Gibco) with 10% FBS (Corning) and 1% streptomycin and penicillin. The cells were maintained under the following conditions: 5% CO_2_ and 95% humidity.

### 2.2. Cell Transfection

Hepatoblastoma cells were transfected with the miR-126 mimic/inhibitor or NC inhibitor (GenePharma, Shanghai, China) by using Lipofectamine 2000 (Thermo Fisher Scientific). Meanwhile, cells were divided into the control (cancer), cancer+inhibitor NC, or cancer+miR-126 inhibitor group.

### 2.3. Isolation and Identification of EVs

Briefly, cells were maintained in DMEM until they reached 80% confluence. The medium was then replaced with the serum-free medium (200 *μ*l). After 48 h of culture, hepatoblastoma cell supernatants were collected by centrifugation (the procedure of centrifugation was as follows: 300 × *g* for 15 min, 2000 × *g* for 15 min, and 10,000 × *g* for 30 min). Subsequently, cell supernatants were filtrated and collected to isolate EVs via ultracentrifugation (120,000 × *g* for 70 min). Centrifugation was performed below 4°C, and washing was performed at intervals with PBS. The content of EV protein was determined by the Bradford method. The particle sizes of EVs were investigated by nanoparticle tracking analysis (NTA), the structure of EVs was observed by transmission electron microscopy (TEM), and the EV markers were detected by western blotting. Meanwhile, the isolation was in accordance with the MISEV guideline.

### 2.4. Nanoparticle Tracking Analysis (NTA)

A total of ~0.3 ml supernatant was loaded into the sample chamber of an LM10 NanoSight (NanoSight, Ltd.), and three videos of either 30 or 60 sec were recorded of each sample. Data analysis was performed using NTA 2.1 software (NanoSight, Ltd.). In NTA, the paths of unlabeled particles acting as point scatterers, undergoing Brownian motion in a 0.25 ml chamber through which a 635 nm laser beam was passed, were determined from a video recording, with the mean squared displacement determined for each possible particle. The diffusion coefficient and sphere-equivalent hydrodynamic radius were subsequently determined using the Stokes-Einstein equation. The sample was measured three times at room temperature.

### 2.5. Transmission Electron Microscopy (TEM)

The EV pellet was incubated for 5 min and subsequently immersed in 2% phosphotungstic acid solution for 1 min. The pellet was fixed using 2.5% glutaraldehyde (pH 7.2) at 4°C overnight. Then, 100 *μ*l suspension was placed on a parafilm sheet and a copper grid coated with carbon was placed onto the drop for 10 s and then removed. The grid was then rinsed 10 times with Milli-Q H_2_O (1 min per rinse) at room temperature. Subsequently, the grid was firstly laid on a drop of uranyl acetate (pH 7.0, 2624, SPI-CHEM, West Chester, PA, USA) at room temperature for 10 min. After rinsing with Milli-Q H_2_O and methylcellulose uranyl (pH 4.0), the grid was incubated at room temperature for 10 min on a drop of methylcellulose uranyl (pH 4.0, M-6385, Sigma-Aldrich).

### 2.6. Bioinformatics Analysis

GSE153089 and GSE75283 datasets containing the miRNA level data for adjacent normal tissues and hepatoblastoma tissues originated from the GEO database. Meanwhile, in GSE153089, 9 hepatoblastoma tissues and 14 normal tissues were involved. In the GSE75283 database, 58 hepatoblastoma tissues and 7 normal tissues were involved. The data were downloaded by using the GEO query and analyzed by using R analysis. ∣logFC | >2 and *P* < 0.05 were identified as the differentially expressed miRNAs. The gene functions in terms of cellular components, biological processes, and molecular functions were identified by GO analysis. Kyoto Encyclopedia of Genes and Genomes (KEGG) was applied to assess the biological pathways. miRTarBase (http://mirtarbase.cuhk.edu.cn/php/index.php) was used to predict the target mRNA of miR-126. In addition, the crosstalk of miRNA-mRNA was pictured by using Cytoscape (https://cytoscape.org/).

### 2.7. Differentiation of BMSCs into Cancer Stem Cells

BMSCs were divided into five groups (control, cancer EVs, NC EVs, miR-126 mimic EVs, and miR-126 inhibitor EVs). BMSCs were treated with cancer EVs, NC EVs, miR-126 mimic EVs, or miR-126 inhibitor EVs for 24 h. Subsequently, the surface markers CD44 (BD Biosciences, New Jersey, USA), CD31 (BD Biosciences), CD45 (BD Biosciences), CD105 (BD Biosciences), CD90 (BD Biosciences), and CD133 (BD Biosciences) were analyzed by flow cytometry (BD Biosciences).

### 2.8. Reverse Transcription-Quantitative PCR (RT-qPCR)

A TRIzol® reagent (Takara, Tokyo, Japan) was used to isolate total RNA from cell lines or tissues. The MicroRNA Reverse Transcription Kit (ELK BioScience, Wuhan, China) or PrimeScript RT Reagent Kit (ELK BioScience, Wuhan, China) was used to reverse-transcribe total RNA into cDNA. In this reverse transcription process, an “A” tail was attached to the miRNAs, increasing their length. Subsequently, the SYBR Premix Ex Taq II Kit (ELK BioScience) was used in RT-qPCR. The protocol was as follows: 2 minutes at 94°C, followed by 35 cycles (30 s at 94°C and 45 s at 55°C). The primer sequences were listed as follows: Oct4, forward 5′-CTCGCTTCGGCAGCACAT-3′ and reverse 5′-AACGCTTCACGAATTTGCGT-3′; miR-126-3p, forward 5′-AGACCACAGTTTGGCAATTGG-3′ and reverse 5′-AGGAGAATCCTGGCACATCG-3′; miR-126-5p, forward 5′-GCAGGACTCACAGCCTTTGG-3′ and reverse 5′-GGCTGGATGTCGGACTTTGT-3′; miR-30b-3p, forward 5′-GCAGGACTCACAGCCTTTGG-3′ and reverse 5′-GGCTGGATGTCGGACTTTGT-3′; miR-30b-3p, forward 5′-GCAGGACTCACAGCCTTTGG-3′ and reverse 5′-GGCTGGATGTCGGACTTTGT-3′; SRY, forward 5′-GCAGGACTCACAGCCTTTGG-3′ and reverse 5′-GGCTGGATGTCGGACTTTGT-3′; IL-1*α*, forward 5′-GCAGGACTCACAGCCTTTGG-3′ and reverse 5′-GGCTGGATGTCGGACTTTGT-3′; IL-6, forward 5′-GCAGGACTCACAGCCTTTGG-3′ and reverse 5′-GGCTGGATGTCGGACTTTGT-3′; TGF-*β*, forward 5′-GCAGGACTCACAGCCTTTGG-3′ and reverse 5′-GGCTGGATGTCGGACTTTGT-3′; *β*-actin, forward 5′-GTCCACCGCAAATGCTTCTA-3′ and reverse 5′-TGCTGTCACCTTCACCGTTC-3′; and U6, forward 5′-GTCCACCGCAAATGCTTCTA-3′ and reverse 5′-TGCTGTCACCTTCACCGTTC-3′. The 2^−*ΔΔ*t^ method was used for quantification. *β*-Actin or U6 was used as an internal control.

### 2.9. Western Blotting

A RIPA lysis buffer (Beyotime) was applied to isolate total protein from cells. The BCA kit (Beyotime) was used to quantify total protein. SDS-PAGE (10%) was applied to separate protein (40 *μ*g per lane), and then proteins were transferred onto PVDF membranes (Thermo Fisher Scientific). After blocked with 5% skimmed milk for 1 h, membranes were incubated with primary antibodies overnight as follows: anti-CD63 (Abcam; ab134045, 1 : 1000), anti-CD9 (Abcam; ab92726, 1 : 1000), anti-CD326 (Abcam; ab239688, 1 : 1000), anti-CD81 (Abcam; ab79559, 1 : 1000), anti-*β*-catenin (Abcam; ab32572, 1 : 1000), anti-ERK (Abcam; ab32537, 1 : 1000), and anti-*β*-actin (Abcam; ab8226, 1 : 1000). After that, the membranes were incubated with secondary antibodies (HRP-conjugated, Abcam; ab7901, 1 : 5000) for 1 h. The ECL kit (Thermo Fisher Scientific) was applied to visualize protein bands. *β*-Actin was used for normalization.

### 2.10. Immunofluorescence (IF)

In brief, cells were fixed in methanol for 20 min at room temperature. Subsequently, 10% goat serum was performed to block the cells for 30 min at room temperature, and then anti-PKH26 (1 : 1000, Abcam) was applied to incubate the cells at 4°C overnight. After that, goat anti-rabbit IgG (1 : 5000, Abcam) was applied to incubate the cells for 1 h at room temperature. Meanwhile, DAPI (Beyotime Institute of Biotechnology) was applied to stain the nuclei for 5 min. Finally, the result was observed by using a microscope (Olympus CX23; Olympus Corporation).

### 2.11. Enzyme-Linked Immunosorbent Assay (ELISA)

The levels of IL-6 (cat no. ab229434) and TGF-*β* (cat no. ab100647) in supernatants of NSCLC cells were assessed by the ELISA detection kit (Abcam). Briefly, the plates (24-well) were pretreated with the primary antibodies (IL-6 or TGF-*β*) overnight. Subsequently, plates were blocked with PBS (5% FBS) for 1 h, and then cells (3 × 10^5^ per well) were added to the plates. After incubation for 2 h, cells were incubated with goat anti-rabbit IgG H&L (ab150077) for 1 h. Cells were treated with 100 *μ*l TMB for 10 min and then were finalized with sulfuric acid (2 M). The absorbance (450 nm) was measured by a microreader.

### 2.12. Immunohistochemical (IHC) Staining

Tumor tissues of mice were fixed overnight and then cut into thick sections (5 *μ*m). The sections were deparaffinized and rehydrated. For antigen retrieval, sections were heated in a microwave (with a sodium citrate buffer). Subsequently, samples were washed with PBS for 5 min. Then, samples were incubated in 3% H_2_O_2_ for 25 min, washed, and incubated in serum for 30 min. After that, anti-*β*-catenin or anti-ERK was applied to incubate the samples at 4°C. Subsequently, the secondary antibody was performed to incubate the samples for 30 min. Finally, diaminobenzidine (DAB) was added and the tissues were observed by using a microscope. All the antibodies were obtained from Abcam.

### 2.13. *In Vivo* Study

BALB/c nude mice (*n* = 9; 6-8 weeks old) were obtained from Vital River (Beijing, China). The protocols for animal care and use of laboratory animals were in accordance with the ethical committee of the Research Ethics Committee of Xiangya Hospital (AF/SQ202104798). Hepatoblastoma cells transfected with the NC inhibitor or hepatoblastoma cells transfected with the miR-126 inhibitor were subcutaneously transplanted in mice (mice were divided into the cancer, cancer+inhibitor NC, or cancer+miR-126 inhibitor group). The tumor volume was investigated once a week as follows: length × width × width. In the end, mice were sacrificed for tumor tissue collection. All *in vivo* experiments were performed in accordance with the National Institutes of Health guide for the care and use of laboratory animals.

### 2.14. Statistical Analysis

Data are presented as the mean ± standard deviation. Comparisons between two groups were analyzed by the unpaired Student's *t*-test. All other experiments were repeated three times. One-way analysis of variance and Tukey's post hoc tests were used for comparisons between ≥3 groups. *P* < 0.05 was considered to indicate a statistically significant difference.

## 3. Results

### 3.1. EVs Were Successfully Isolated from Tumor Tissues

**Figure 1:**
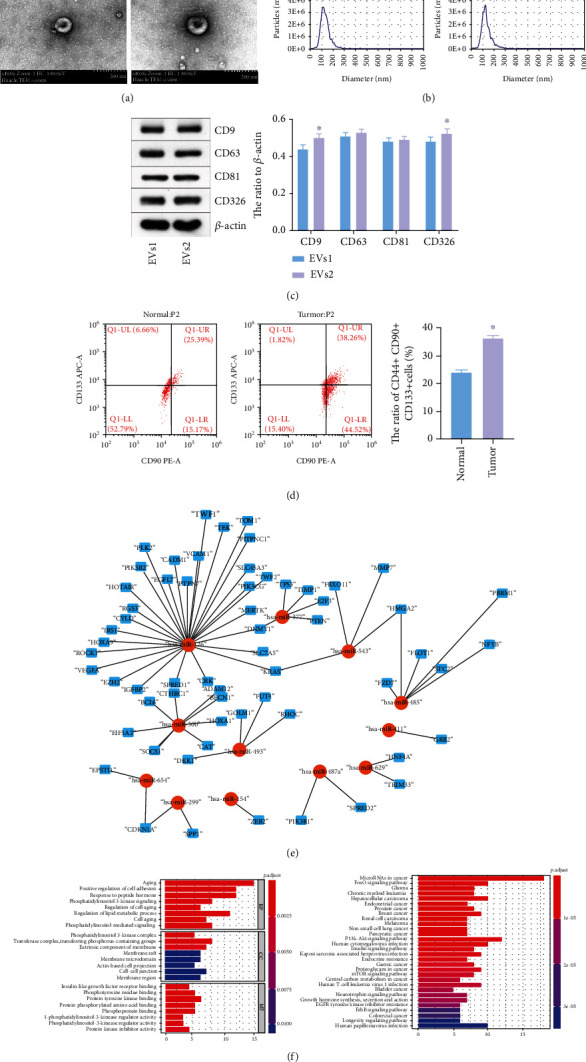
EVs were successfully isolated from hepatoblastoma tissues. (a) The separation efficiency of EVs was examined by TEM. (b) The particle sizes of EVs derived from cancer and normal tissues were measured by NTA. (c) CD9, CD63, CD81, and CD326 levels in EVs1 and EVs2 were assessed by western blot. *β*-Actin was applied for normalization. ^∗^*P* < 0.05 compared to EVs1. (d) The ratio of CD44+ CD90+ CD133+ cells in normal and tumor tissues was assessed by flow cytometry. ^∗^*P* < 0.05 compared to normal tissue. (e) The miRNA-mRNA network in hepatoblastoma was presented. (f) The target of overlapped miRNAs was subjected to GO and KEGG analyses.

Firstly, we aimed to isolate the EVs from hepatoblastoma and adjacent normal tissues, and then TEM and NTA were used to identify the EVs. As indicated in Figure 1(a), typical saucer-like particles were observed by TEM. NTA exhibited that the diameter of EVs was 30 to 150 nm (Figure 1(b)). In addition, CD9 and CD326 levels in EVs2 were significantly higher, compared with those in EVs1. However, the protein levels of CD63 and CD81 in EVs1 were almost the same as those in EVs2 (Figure 1(c)). These results indicated that EVs were successfully isolated from tumor tissues. The ratio of CD44+ CD90+ CD133+ cells in tumor tissues was notably higher, compared with that in normal tissues (Figure 1(d)). Meanwhile, in the GSE75283 dataset (DS), 96 miRNAs were differentially expressed (43 were downregulated, and 53 were upregulated) (Supplementary Figure 1A). In GSE153089 DS, 46 miRNAs were differentially expressed (41 were downregulated, and 5 were upregulated) (Supplementary Figure 1B). Among these differentially expressed miRNAs, 83 were commonly downregulated, whereas 33 were commonly upregulated (Supplementary Figure 1C). Moreover, 13 miRNAs were overlapped between these upregulated and downregulated miRNAs (Supplementary Figure 1C). The data of GO and KEGG indicated these overlapped miRNAs were closely correlated with aging and cancer growth (Figures 1(e) and 1(f)). Taken together, EVs were successfully isolated from tumor tissues.

### 3.2. miR-126 Was Upregulated in Cancer Cells

**Figure 2:**
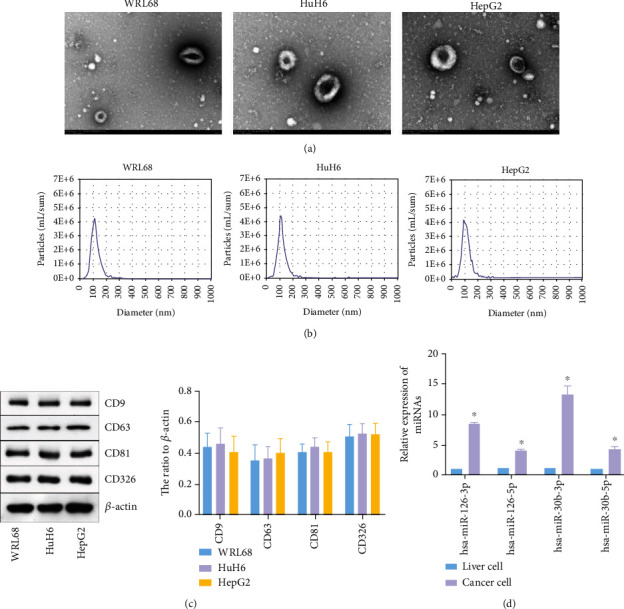
miR-126 was upregulated in cancer cells. (a) The separation efficiency of EVs was examined by TEM. (b) The particle sizes of EVs derived from hepatoblastoma and normal liver cells were measured by NTA. (c) The expressions of CD9, CD63, CD81, and CD326 in hepatoblastoma cells were assessed by western blotting. *β*-Actin was applied for normalization. (d) The expressions of miR-126-3p, miR-126-5p, miR-30b-3p, and miR-30b-5p in liver or cancer cells were detected by RT-qPCR. ^∗^*P* < 0.05 compared to liver cells.

To detect the efficiency of EV isolation, TEM and NTA were performed. As indicated in Figure 2(a), typical saucer-like particles were observed by TEM. NTA showed that the diameter of EVs was 30 to 150 nm (Figure 2(b)). In addition, WB showed positive expression of CD9, CD81, CD63, and CD326 proteins in the extract (Figure 2(c)). All the results suggested that EVs were successfully isolated from cancer cells. Since miR-126-3p, miR-126-5p, miR-30b-3p, and miR-30b-5p were associated with cancer growth [29–31], these miRNAs were selected for subsequent analysis. The data revealed the expressions of miR-126-3p, miR-126-5p, miR-30b-3p, and miR-30b-5p in cancer cells were significantly higher than those in normal liver cells (Figure 2(d)). In summary, miR-126 was upregulated in cancer cells.

### 3.3. EVs Promoted the Differentiation of BMSCs into Cancer Stem Cells

**Figure 3:**
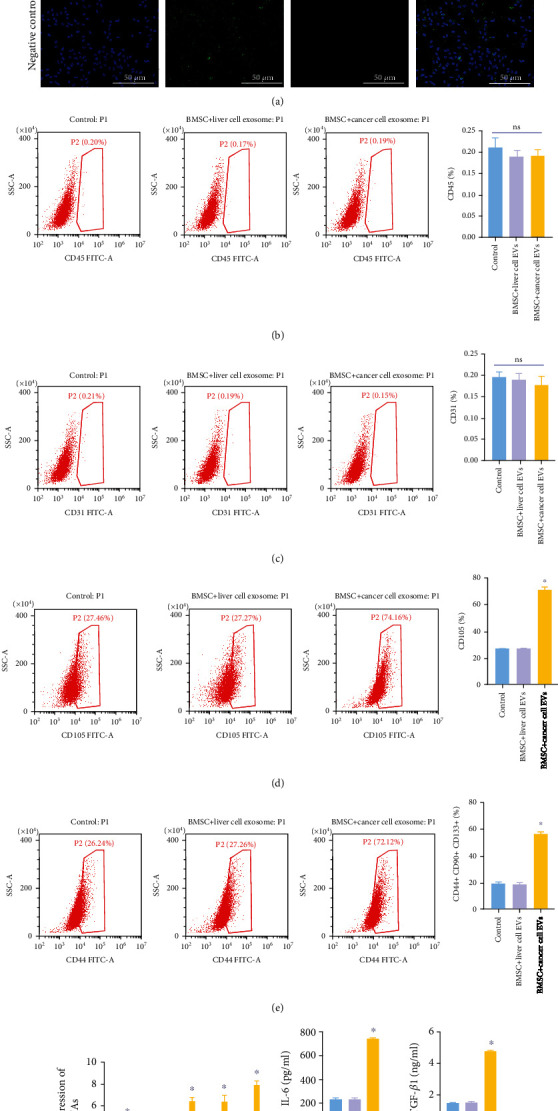
EVs promoted the differentiation of BMSCs into cancer stem cells. EVs derived from tumor or normal tissues were cocultured with BMSCs for 48 h. Then, (a) the EV uptake was observed by IF staining. Scale bar: 100 *μ*m. 100x magnification. (b) Flow cytometry was performed to test the ratio of CD44 in BMSCs. (c) Flow cytometry assessed the ratio of CD31 in BMSCs. (d) Flow cytometry tested the ratio of CD105 in BMSCs. (e) Flow cytometry assessed the ratio of CD44+ CD90+ CD133+ cells in BMSCs. (f) RT-qPCR evaluated Oct4, SRY, IL-1*α*, IL-6, and TGF-*β*1 levels in BMSCs. (g) ELISA was performed to test the content of IL-6 and TGF-*β*1 in supernatants of BMSCs. ^∗^*P* < 0.05 compared to the control. ns indicated no significant change.

To investigate the function of EVs in BMSCs, BMSCs were cocultured with EVs. As indicated in Figure 3(a), the expression of PKH67 (the label of EVs) was significantly upregulated in BMSCs. In addition, the ratio of CD31 and CD45 was limitedly affected by EVs, while EVs derived from tumor cells significantly increased the ratio of CD105 and CD44+ CD90+ CD133+ in BMSCs (Figures 3(b)–3(e)). Moreover, the expressions of IL-1*α*, SRY, Oct4, IL-6, and TGF-*β* in BMSCs were markedly increased by cancer cell-derived EVs (Figure 3(f)). Consistently, the contents of IL-6 and TGF-*β*1 in BMSC supernatants were significantly upregulated in the presence of EVs derived from hepatoblastoma cells (Figure 3(g)). To sum up, EVs promoted the differentiation of BMSCs into cancer stem cells.

### 3.4. Downregulation of miR-126 Inhibited the Tumor Growth of Hepatoblastoma

**Figure 4:**
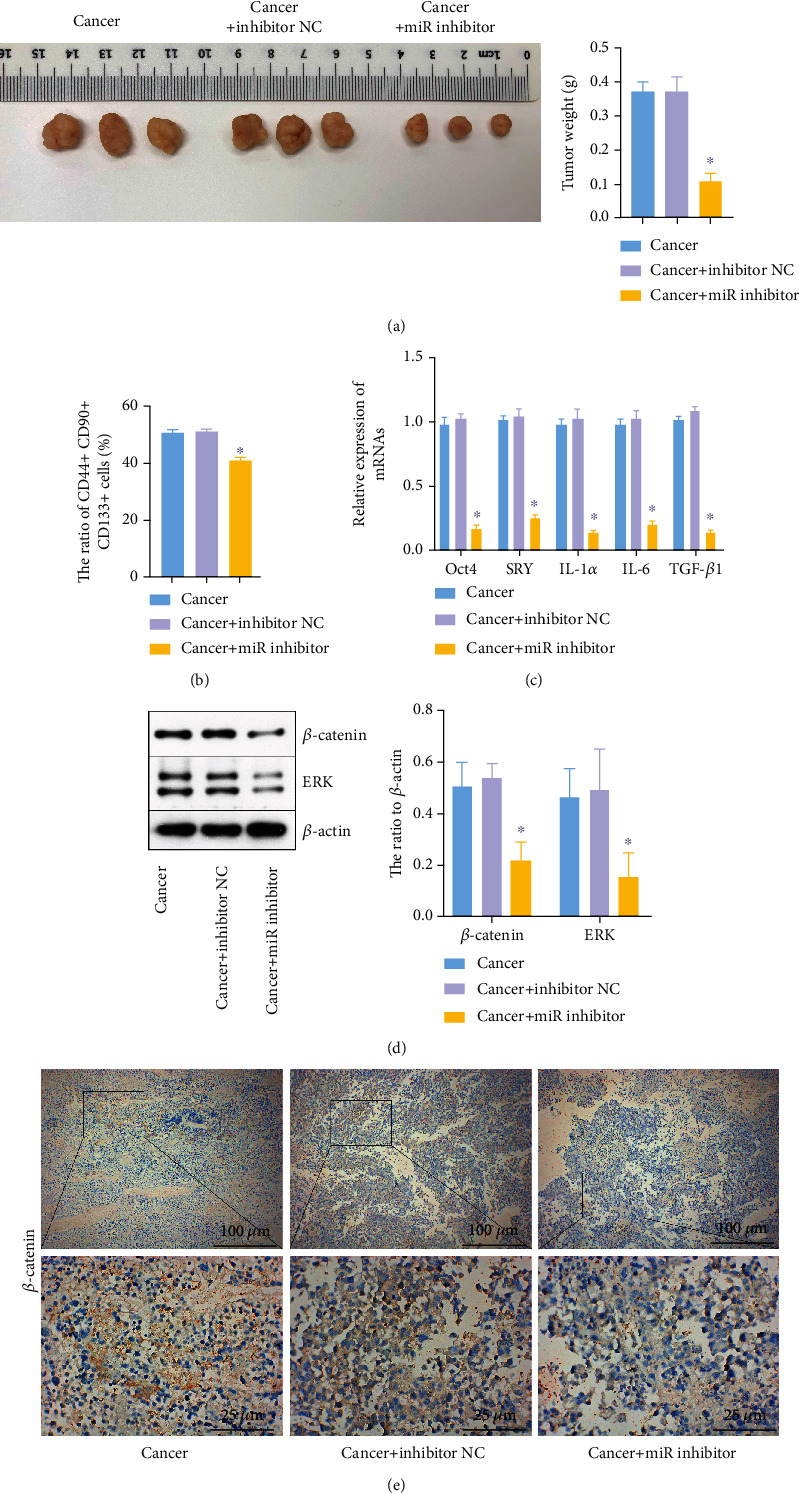
The miR-126 inhibitor inhibited the tumor growth of hepatoblastoma. Mice were divided into the cancer, cancer+inhibitor NC, and cancer+miR inhibitor groups. (a) The tumor tissues of mice were collected, pictured, and weighted after mice were sacrificed. (b) The ratio of CD44+ CD90+ CD133+ cells was measured by flow cytometry. (c) Oct4, SRY, IL-1*α*, IL-6, and TGF-*β*1 levels in tissues were investigated by RT-qPCR. (d) *β*-catenin and ERK levels in tissues of mice were assessed by western blotting. *β*-Actin was applied for normalization. (e) The expressions of *β*-catenin were assessed by IHC staining. ^∗^*P* < 0.05 compared to cancer.

To investigate the function of miR-126 in hepatoblastoma, a xenograft mouse model was established. As shown in Figure 4(a), downregulation of miR-126 significantly decreased the tumor size and weight of hepatoblastoma. In addition, the miR-126 inhibitor obviously decreased the ratio of CD44+ CD90+ CD133+ cells, as well as the levels of IL-1*α*, SRY, Oct4, IL-6, and TGF-*β* in tumor tissues of mice (Figures 4(b) and 4(c)). Meanwhile, the protein levels of *β*-catenin and ERK in tumor tissues of mice were notably reduced by the miR-126 inhibitor (Figures 4(d) and 4(e)). Altogether, downregulation of miR-126 inhibited the tumor growth of hepatoblastoma.

### 3.5. EVs with Downregulated miR-126 Significantly Inhibited the Differentiation of BMSCs into Cancer Stem Cells

**Figure 5:**
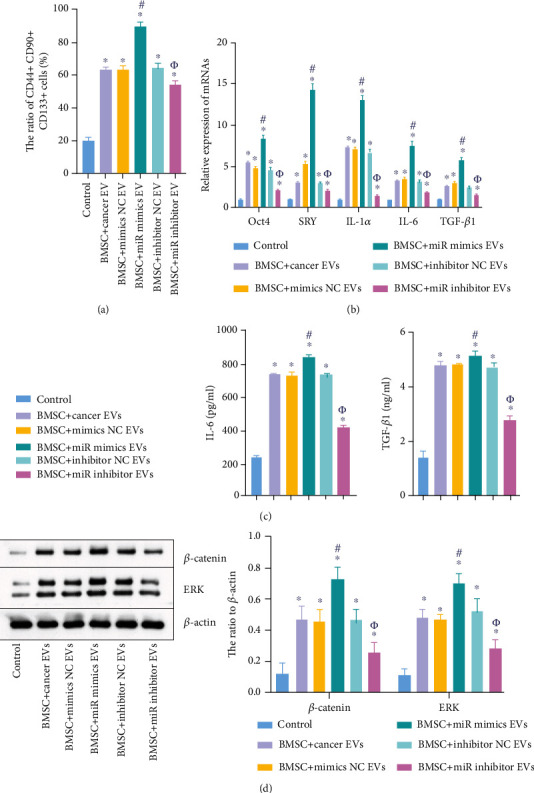
EVs with downregulated miR-126 significantly inhibited the differentiation of BMSCs into cancer stem cells. BMSCs were divided into the following groups: control, BMSC+miR mimic EVs, BMSC+cancer EVs, BMSC+inhibitor NC EVs, BMSC+mimic NC EVs, and BMSC+miR inhibitor EVs. (a) BMSCs were cocultured with cancer EVs, mimic NC EVs, miR mimic EVs, inhibitor NC EVs, or miR inhibitor EVs. Then, the ratio of CD44+ CD90+ CD133+ cells in BMSCs was assessed by flow cytometry. (b) Oct4, SRY, IL-1*α*, IL-6, and TGF-*β*1 levels in BMSCs were assessed by RT-qPCR. (c) The content of IL-6 and TGF-*β*1 in BMSC supernatants was investigated by ELISA. (d) The protein levels of *β*-catenin and ERK in BMSCs were tested by western blot. *β*-Actin was applied for normalization. ^∗^*P* < 0.05 compared to the control. ^#^*P* < 0.05 compared to BMSC+cancer EVs. *^Φ^P* < 0.05 compared to BMSC+inhibitor NC EVs.

To further investigate the function of EVs in the differentiation of BMSCs, flow cytometry was performed. As revealed in Figure 5(a), EVs derived from cancer cells significantly upregulated the ratio of CD44+ CD90+ CD133+ cells in BMSCs, while this phenomenon was reversed by EVs with downregulated miR-126 and aggravated by miR-126-overexpressed EVs. Meanwhile, EV-induced upregulation of IL-1*α*, SRY, Oct4, IL-6, and TGF-*β* was further enhanced by exosomal miR-126, while EVs with downregulated miR-126 exerted the opposite effect (Figure 5(b)). Consistently, EVs carrying the miR-126 inhibitor notably suppressed the secretion of IL-6 and TGF-*β* in BMSCs (Figure 5(c)). Mechanistically, the expressions of *β*-catenin and ERK in BMSCs were significantly upregulated by EVs derived from cancer cells, which were partially reversed by miR-126 inhibitor EVs (Figure 5(d)). Conversely, miR-126 mimic EVs further increased the effect of EVs alone on these two proteins (Figure 5(d)). Taken together, EVs with downregulated miR-126 significantly inhibited the differentiation of BMSCs into cancer stem cells.

## 4. Discussion

Hepatoblastoma is reported to be the frequent liver tumor, which seriously influences majorly children (less than 4 years old) [32]. Although the incidence of hepatoblastoma has been increasing significantly in ten years, hepatoblastoma is a pediatric malignant tumor which accounts for 1.5 cases per million [33]. Nowadays, the treatments (chemotherapy and surgical resection) have led to increasing the survival rate (up to 80%) of all patients with hepatoblastoma [34]. Nevertheless, the prognosis of patients with diagnosed advanced hepatoblastoma is still not ideal. Moreover, patients suffering the treatments (chemotherapy and surgery) can suffer constant side effects which results from immunosuppression and chemotherapy [35]. The relatively poor prognosis for patients with hepatoblastoma is due to the limited effective means of early diagnosis [36]. Presently, this disease was diagnosed majorly based on imaging, clinical symptoms, and alpha-fetoprotein expressions [37]. Thus, new targets must be found to develop the methods of efficient diagnoses and strategies of therapy against hepatoblastoma. In this research, we performed bioinformatics analysis according to the volcanic map method for hepatoblastoma using various miRNAs. In addition, two independent datasets were also used to identify the differentially expressed miRNAs. Then, these methods might lead to developing hepatoblastoma diagnosis. Furthermore, GO and KEGG analyses were also performed, and the data revealed that the identified miRNAs were mainly involved in aging and cancer growth. As a result, miR-126 was identified by cross-referencing our investigations, which may provide new strategies.

It has been reported that EVs play a crucial role in cancer development. For instance, Xie et al. suggested that exosomal miR-193a-3p derived from hypoxic BMSCs could promote lung cancer cell invasion through regulation of STAT3 and the EMT process [38]; Wu et al. showed that exosomal miR-126 derived from BMSCs could inhibit the tumorigenesis of pancreatic cancer through targeting ADAM9 [39]. In this research, we found miR-126 was upregulated in hepatoblastoma, and EVs with downregulated miR-126 could promote the differentiation of BMSCs into cancer stem cells. Thus, our study firstly explored the mechanism by which exosomal miRNA mediates the differentiation of BMSCs, suggesting a novel strategy for hepatoblastoma treatment. Meanwhile, Li et al. found that exosomal miR-126 could attenuate the development of NSCLC through inhibiting the expression of ITGA6 [40], and this result was the opposite of our study. This discrepancy might be due to the different tumor types.

It has been reported that CD31, CD45, CD44, CD105, and CD133 are key markers during the differentiation of BMSCs [38, 41]. In BMSC differentiation, CD105 expression is upregulated, and the CD44+ CD90+ CD133+ cells' ratio is often increased [42]. In this study, we found that the expression of CD105 and the ratio of CD44+ CD90+ CD133+ cells were upregulated by EVs derived from cancer cells. Thus, our data were consistent with the previous data. On the other hand, IL-1*α*, SRY, Oct4, IL-6, and TGF-*β* have been reported to play vital roles in osteogenic differentiation [43, 44]. These four factors are considered to be upregulated when osteogenic differentiation happens [45, 46]. Consistently, our data further confirmed that IL-1*α*, SRY, Oct4, IL-6, and TGF-*β* played important roles in osteogenic differentiation.

It has been demonstrated that miR-126 plays a vital role in cancer development. For example, Ma et al. showed that exosomal miR-126 might play a vital role in colorectal cancer [47]; Li et al. found that exosomal miR-126 derived from NSCLC serum could suppress the progression of NSCLC through inactivation of ITGA6 [40]. Our study found that the miR-126 inhibitor could attenuate the growth of hepatoblastoma, which firstly found the function of miR-126 in hepatoblastoma. Meanwhile, other miRNAs are also involved in the progression of hepatoblastoma. For example, miR-203 could regulate angiogenesis in hepatoblastoma [16]; Cui et al. found that miR-186 could inhibit the tumorigenesis of hepatoblastoma [25]. Thus, more miRNAs related to hepatoblastoma progression remain unexplored.

Wnt signaling has been considered an important player in cell differentiation and growth [48, 49], and *β*-catenin is the core protein in Wnt signaling [50]. In this research, we found that EVs with downregulated miR-126 could inhibit the activation of *β*-catenin. Cui et al. found MSR1 could promote the differentiation of BMSCs and M2-like polarization by activation of the PI3K/AKT/GSK3*β*/*β*-catenin pathway [25]. Thereby, our study was similar to this previous research, suggesting that Wnt/*β*-catenin signaling is a crucial mediator during the differentiation of BMSCs into cancer stem cells. Otherwise, some reports indicated that AKT and p53 pathways were also involved in the differentiation of BMSCs [51, 52]. Therefore, more signaling pathways other than Wnt are needed to be investigated in the future.

Of course, some limitations exist in this research as follows: (1) the target mRNAs of miR-126 need to be further explored; and (2) more mechanisms by which exosomal miR-126 regulates the differentiation of BMSCs remain to be further explored. Therefore, more investigations are needed in the coming future.

## 5. Conclusion

In conclusion, exosomal miR-126 derived from hepatoblastoma cells promotes the tumorigenesis of hepatoblastoma through inducing the differentiation of BMSCs into cancer stem cells. Therefore, our findings would shed new light on exploring the new methods for hepatoblastoma treatment.

## Data Availability

Data generated for the current study are available from the corresponding author on reasonable request.

## Supplementary Materials

Supplementary Materials Supplementary Figure 1: the differentially expressed miRNAs between the hepatoblastoma and adjacent normal tissues. (A) miRNAs differentially expressed in hepatoblastoma assessed in the GSE153089 dataset were listed in volcano plots. Red indicates a higher expression level, while blue indicates a lower expression level. (B) Volcano plots illustrating the miRNAs differentially expressed in hepatoblastoma detected in the GSE75283 dataset. Red indicates higher expressed miRNAs while blue represents lower expressed miRNAs. (C) The overlap among the differentially expressed miRNAs between the GSE153089 and GSE75283 datasets was presented in the Venn diagram.
