# Supplementary figures and images for: miR-126 in Extracellular Vesicles Derived from Hepatoblastoma Cells Promotes the Tumorigenesis of Hepatoblastoma through Inducing the Differentiation of BMSCs into Cancer Stem Cells
Source: J Immunol Res. 2021 Oct 29;2021:6744715. doi: 10.1155/2021/6744715 (PMC8570887; doi:10.1155/2021/6744715)

A

Differentially Expressed miRNA of GSE153089

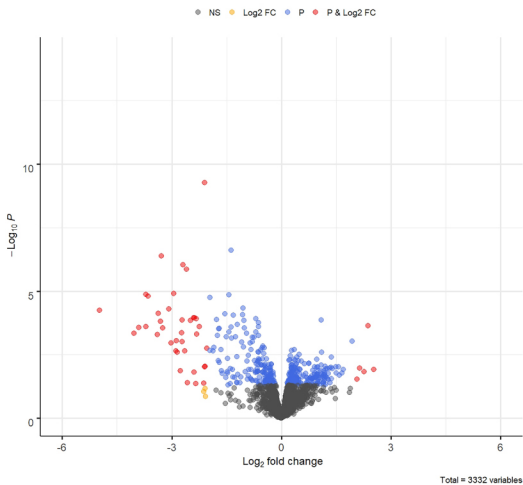

B

Differentially Expressed miRNA of GSE75283

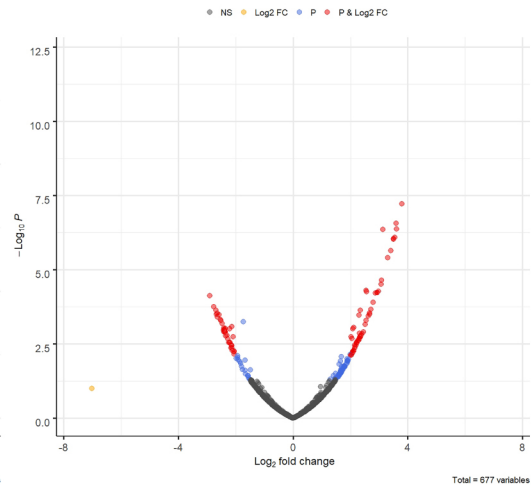

C

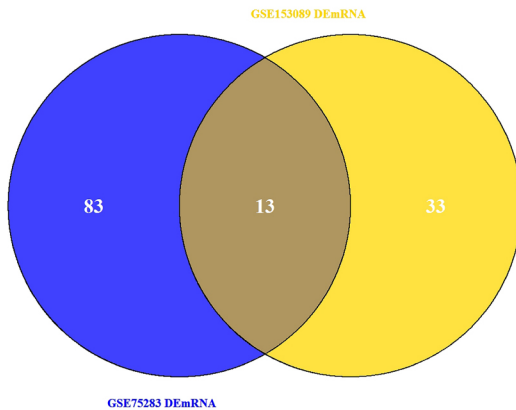

Supplement: Supplementary Materials — Supplementary Figure 1: the differentially expressed miRNAs between the hepatoblastoma and adjacent normal tissues. (A) miRNAs differentially expressed in hepatoblastoma assessed in the GSE153089 dataset were listed in volcano plots. Red indicates a higher expression level, while blue indicates a lower expression level. (B) Volcano plots illustrating the miRNAs differentially expressed in hepatoblastoma detected in the GSE75283 dataset. Red indicates higher expressed miRNAs while blue represents lower expressed miRNAs. (C) The overlap among the differentially expressed miRNAs between the GSE153089 and GSE75283 datasets was presented in the Venn diagram. [file 6744715.f1.pdf]
